# Monocyte-to-lymphocyte ratio is a promising biomarker in patients initially receiving hemodialysis

**DOI:** 10.3389/fneph.2025.1638388

**Published:** 2025-09-23

**Authors:** Aihua Xie, Anna Tang, Man Yang, Yuwan Xiong, Jieshan Lin

**Affiliations:** ^1^ Department of Nephrology, Blood Purification Center, Zhongshan City People’s Hospital, Zhongshan, China; ^2^ Department of Nephrology, Guangdong Provincial People’s Hospital (Guangdong Academy of Medical Sciences), Southern Medical University, Guangzhou, China

**Keywords:** monocyte-to-lymphocyte ratio, hemodialysis, mortality, biomarker, inflammation

## Abstract

**Aim:**

Inflammation is very common among dialysis patients and can lead to an increase in morbidity and mortality. Monocyte-to-lymphocyte ratio (MLR) can serve as a reliable predictor of long-term survival in hemodialysis patients. However, few studies have addressed the role of MLR in patients initially receiving hemodialysis (within 3 months). In this study, we aimed to examine the association between MLR and the risk of cardiovascular and all-cause mortality in patients initially receiving hemodialysis.

**Methods:**

In this study, a total of 216 patients newly receiving hemodialysis for at least 3 months were recruited. The associations between MLR and cardiovascular diseases (CVD) and all-cause mortality were assessed by multivariable Cox models.

**Results:**

A total of 216 patients were included (mean age 57.65 ± 15.68 years, 42.13% male patients). Patients were divided into the low MLR group (<0.49) and the high MLR group (≥0.49). The levels of neutrophil and serum iron and the number of deaths were significantly higher in the high MLR group (*P* < 0.05). Spearman’s analysis showed that MLR was positively correlated with BUN (*R* = 0.210, *P* = 0.002), WBC (*R* = 0.178, *P* = 0.009), and neutrophil (*R* = 0.237, *P* < 0.001). Kaplan–Meier analysis showed that patients in the low MLR group present longer survival (64.08 ± 2.30 vs. 51.07 ± 3.12 months, *P* < 0.001). Multivariate Cox regression analysis showed that age, diabetes, and MLR (all *P* < 0.05) were factors significantly associated with a higher risk of CVD and all-cause mortality.

**Conclusions:**

Our results showed that high MLR values are an independent risk factor for CVD and all-cause mortality in patients initially receiving hemodialysis, especially in the elderly and those with a history of diabetes.

## Introduction

Chronic kidney disease (CKD) is a global public health problem due to its escalating prevalence and the rising number of people receiving dialysis. Hemodialysis is the most common modality of kidney replacement therapy, with a worldwide prevalence of more than 85% (1). Dialysis patients have poor clinical outcomes, and the 5-year survival rate after the initiation of maintenance dialysis is approximately 40% (2). Cardiovascular diseases (CVDs) and non-cardiovascular factors, especially infection-related complications, are the main causes of the increased premature mortality rate observed in the hemodialysis population, driven by immune dysfunction and chronic inflammatory states. After initiation of maintenance dialysis, CVDs account for approximately 50% of the total mortality rate, while infections account for 20% (3).

Dialysis is bidirectionally negatively correlated with persistent inflammation. A variety of risk factors in hemodialysis patients can lead to persistent inflammatory responses, such as reduced clearance of inflammatory factors, oxidative stress, uremia, malnutrition, dyslipidemia, and infections (4). Sustained inflammatory responses in hemodialysis patients contribute to a spectrum of complications through mechanisms involving immune dysregulation, oxidative stress, and multi-organ damage, like CVD, anemia, malnutrition, infections, and bone mineral disorders (5). Therefore, the monitoring of inflammation-related markers holds significant clinical value for assessing prognosis and conducting early intervention in hemodialysis patients.

Traditional inflammatory markers, including IL-1β, IL-6, IFN-γ, CRP, and TNF-α, have disadvantages such as high cost, complex technological process, and poor disease specificity, making them difficult to be widely applied in clinical practice (6). The ratios of different blood cell components, including monocyte-to-lymphocyte ratio (MLR), neutrophil-to-lymphocyte ratio (NLR), platelet-to-lymphocyte ratio (PLR), and platelet-to-albumin ratio (PAR), are inexpensive and easy to obtain clinically and have been proven to be novel prognostic indicators for CKD. Previous studies have reported that MLR was a better predictor of CVD and all-cause mortality in hemodialysis and peritoneal dialysis patients (7). Because the mortality rate of patients after the initiation of maintenance dialysis is relatively high, this study aimed to examine the association between MLR and the risk of CVD and all-cause mortality in patients initially receiving hemodialysis.

## Materials and methods

### Patients

This was a single-center retrospective study that included a total of 216 patients newly receiving hemodialysis for at least 3 months between January 2018 and December 2019 in our hospital. The inclusion criteria were as follows: adults with end-stage renal disease (ESRD) requiring first-time hemodialysis. The study excluded the following populations: 1) younger than 18 years; 2) patients with malignancy, acute kidney injury, acute heart failure, active systemic infections, autoimmune diseases, or hematological diseases; 3) patients with incomplete clinical test results; and 4) patients undergoing renal replacement therapy.

### Clinical outcome

The outcome of this study was CVD and all-cause mortality. All patients were followed until death, cessation of hemodialysis, or the end of the study period (31 December 2024).

### Data collection

All baseline data were collected 1–3 months following an initial 3-month period of hemodialysis treatment. For all patients, we recorded demographic data, including sex, age, history of hypertension and diabetes, and primary cause of ESKD. Clinical and biochemical data included serum creatinine (SCr), blood urea nitrogen (BUN), carbon dioxide combining power (CO_2_CP), serum albumin, β2-microglobulin, uric acid, cystatin, fasting blood glucose, HbA1c, low-density lipoprotein (LDL), cholesterol, triglyceride, white blood cell (WBC), neutrophil, lymphocytes, monocyte, platelet and hemoglobin levels, serum iron, serum potassium (K), calcium (Ca), phosphorus (P), and intact parathyroid hormone (iPTH). MLR, NLR, and PLR were calculated by dividing monocytes, neutrophils, and platelets by lymphocytes, respectively. PAR was calculated as the platelet-to-albumin ratio.

### Statistical analysis

**Figure 1:**
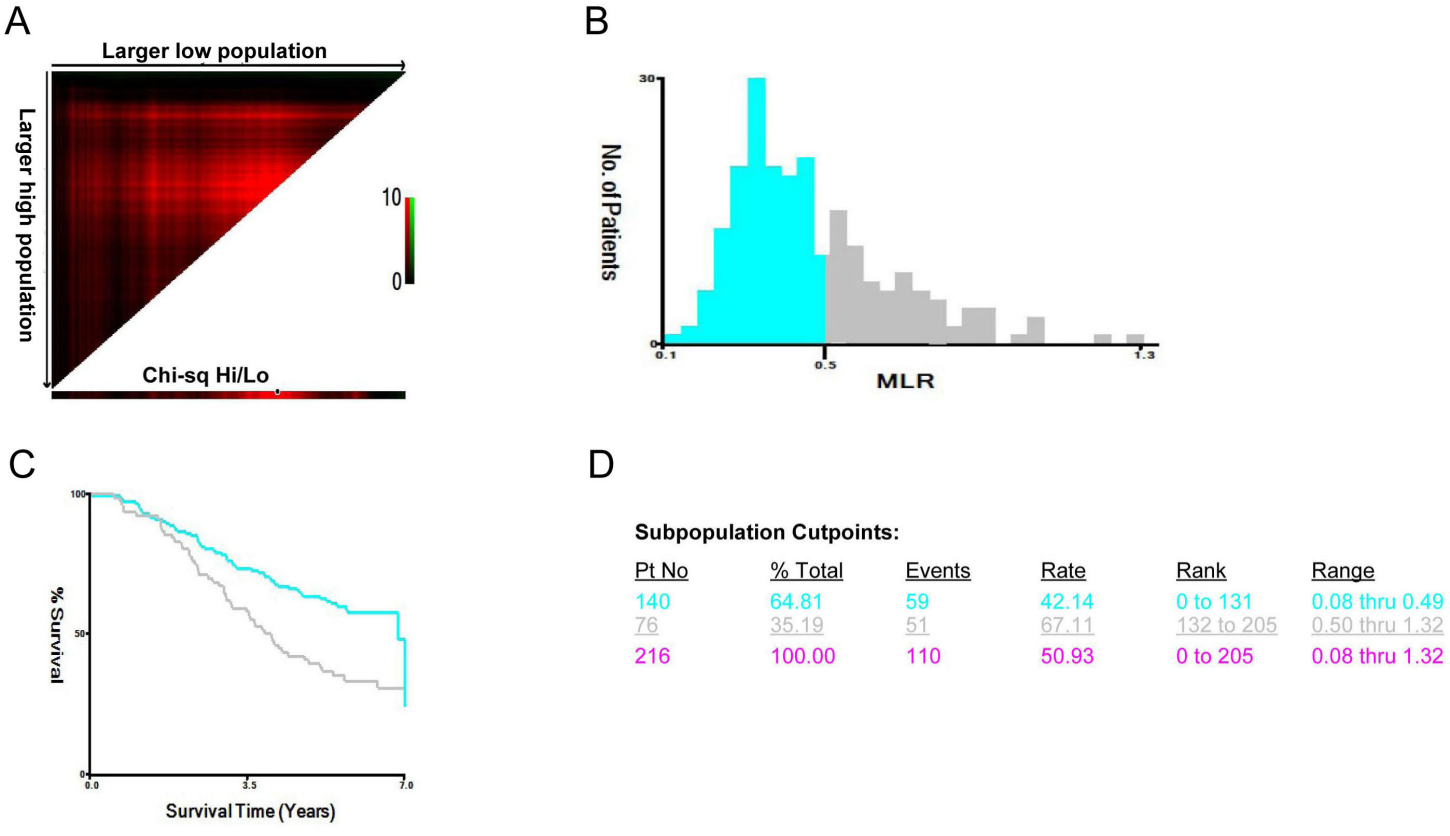
The optimal cutoff points of MLR. **(A)** The X-tile plot reveals the distribution of the population. The best MLR cutoff values are shown in the histogram **(B)** and Kaplan–Meier plots **(C)** for the entire cohort. **(D)** The subpopulation cutoff points of the population. The optimal cutoff point of MLR was 0.49 based on the outcome, and we divided the cases into low (*n* = 140 cases) and high (*n* = 76 cases) populations based on a cutoff point.

All statistical analyses were performed using SPSS version 20.0 (Chicago, IL, USA). The data were presented as frequency (percentage) for categorical variables, mean ± standard deviation for normally distributed continuous variables, and median (Q1–Q3) for non-normally distributed data. Continuous variables were analyzed by the Wilcoxon rank-sum test. The correlations between MLR and clinical data were performed using Spearman’s test. The X-tile software version 3.6.1 (Yale University, New Haven, USA) was used to determine the optimal cutoff points of MLR based on the outcome (8). Through X-tile analysis of survival outcomes, MLR demonstrated a continuous prognostic relationship. The analytical cohort was randomly allocated into equally sized training and validation sets for robust evaluation. **Figure 1** shows MLR divided at the optimal cutoff point, as defined by the most significant (brightest pixel) on the plot (0.49). The cutoff point highlighted by the black/white circle in the left panels is shown on a histogram of the entire cohort and on a Kaplan–Meier plot (right panels; low subset = blue, high subset = gray). Receiver operating characteristic (ROC) curve analysis was performed to evaluate the predictive accuracy of the MLR for all-cause mortality in our patients, with AUC values interpreted as 0.5 (no discrimination) to 1.0 (perfect discrimination). The univariate and multivariate Cox proportional hazards models were used to examine the associations between clinical data and cardiovascular and all-cause mortality. A *P*-value <0.05 was considered statistically significant.

## Results

### Patient and demographic details

A total of 216 patients initially receiving hemodialysis for at least 3 months were included in the final analysis. The mean (± SD) age was 57.65 ± 15.68 years, and 42.13% were male patients. Of the 216 patients, 141 patients (65.28%) had hypertension and 89 (41.20%) had diabetes. The optimal cutoff point of MLR was 0.49 based on the outcome. Patients were divided into the low MLR group (<0.49) and the high MLR group (≥0.49). There were 140 patients in the low MLR group and 76 patients in the high MLR group. The levels of neutrophil and serum iron and the number of deaths were significantly higher in the high MLR group (*P* < 0.05). There was no statistically significant difference among the groups in other laboratory data. The baseline characteristics of the patients are shown in **Table 1**. We found that neutrophil, serum iron, PCT, and the number of deaths were higher in the high MLR group (*P* < 0.05).

**Table 1:** Clinical characteristics and laboratory parameters of the study population.

Characteristics	Low MLR group	High MLR group	P value	Reference interval
Number of cases	140	76		
Age, years	56.81±15.46	59.75±16.01	0.865	
Gender (M/F)	77/63	48/28		
Hypertension	63.57%	68.42%	0.182	
SCr (µmol/L)	882.64±329.78	890.97±309.05	0.624	45.0-84.0
BUN (mmol/L)	24.31±6.89	26.69±8.01	0.071	2.90-8.20
CO2 (mmol/L)	19.94±4.16	19.61±3.68	0.874	23.0-29.0
Albumin (g/L)	37.15±4.94	37.26±4.28	0.144	40.0-55.0
β2-MG	25.67±10.19	25.79±5.87	0.249	1.00-3.00
Uric acid (µmol/L)	462.73±118.92	451.99±142.15	0.082	155-357
Cystatin (mg/L)	6.61±2.45	7.08±2.54	0.488	0.60-1.55
Fasting blood glucose (mmol/L)	5.86±2.57	6.32±2.75	0.341	3.89-6.11
HbA1c	6.23±1.46	7.08±2.54	0.755	3.8-6.2
LDL (mmol/L)	2.51±1.12	2.35±0.94	0.728	<3.36
Cholesterol (mmol/L)	4.43±1.40	5.31±8.30	0.119	<5.18
Triglyceride (mmol/L)	1.58±0.92	1.42±1.09	0.595	<1.70
WBC (10^9^/L)	6.77±1.96	7.22±2.09	0.537	3.69-9.16
Neutrophil (10^9^/L)	4.41±1.68	5.25±2.50	<0.001*	2.00-7.00
Lymphocytes (10^9^/L)	1.46±0.49	0.93±0.44	0.05	0.80-4.00
Monocytes (10^9^/L)	0.48±0.18	0.62±0.27	0.108	0.12-1.00
Platelets (10^9^/L)	211.19±77.14	219.80±90.53	0.197	101.0-320.0
Haemoglobin (g/L)	92.52±18.77	89.03±19.03	0.733	113.0-151.0
Serum iron (µmol/L)	9.71±10.74	12.14±26.78	0.045*	9.0-27.0
hs-CRP (mg/L)	10.33±18.99	19.11±39.20	0.308	0-5.0
PCT (ng/ml)	0.75±0.82	5.97±18.70	0.043*	0-0.05
IL-6 (pg/ml)	29.53±24.84	28.44±10.97	0.945	0-7.0
MLR (before dialysis)	0.54±0.35	0.84±0.69	<0.001*	
MLR (after dialysis)	0.34±0.09	0.70±0.18	<0.001*	
K (mmol/L)	4.93±1.69	4.86±0.95	0.347	3.50-5.30
Ca (mmol/L)	2.43±3.35	2.11±0.27	0.224	2.08-2.60
P (mmol/L)	1.97±0.68	2.05±0.81	0.054	0.96-1.62
iPTH (pg/ml)	266.80±295.64	249.47±275.53	0.164	15.0-65.0
Number of deaths	59 (42.14%)	51 (67.11%)	0.005*	

SCr, serum creatinine; BUN, blood urea nitrogen; CO2, carbon dioxide combining power; β2-MG, β2-microglobulin; HbA1c, glycated haemoglobin; LDL, low density lipoprotein; WBC, white blood cell; hs-CRP, high-sensitivity C-reactive protein; PCT, procalcitonin; IL-6, Interleukin 6; MLR, monocyte-to-lymphocyte ratio; iPTH, intact parathyroid hormone. *p<0.05. Values in bold represent statistically significant differences (p<0.05).

**Figure 2:**
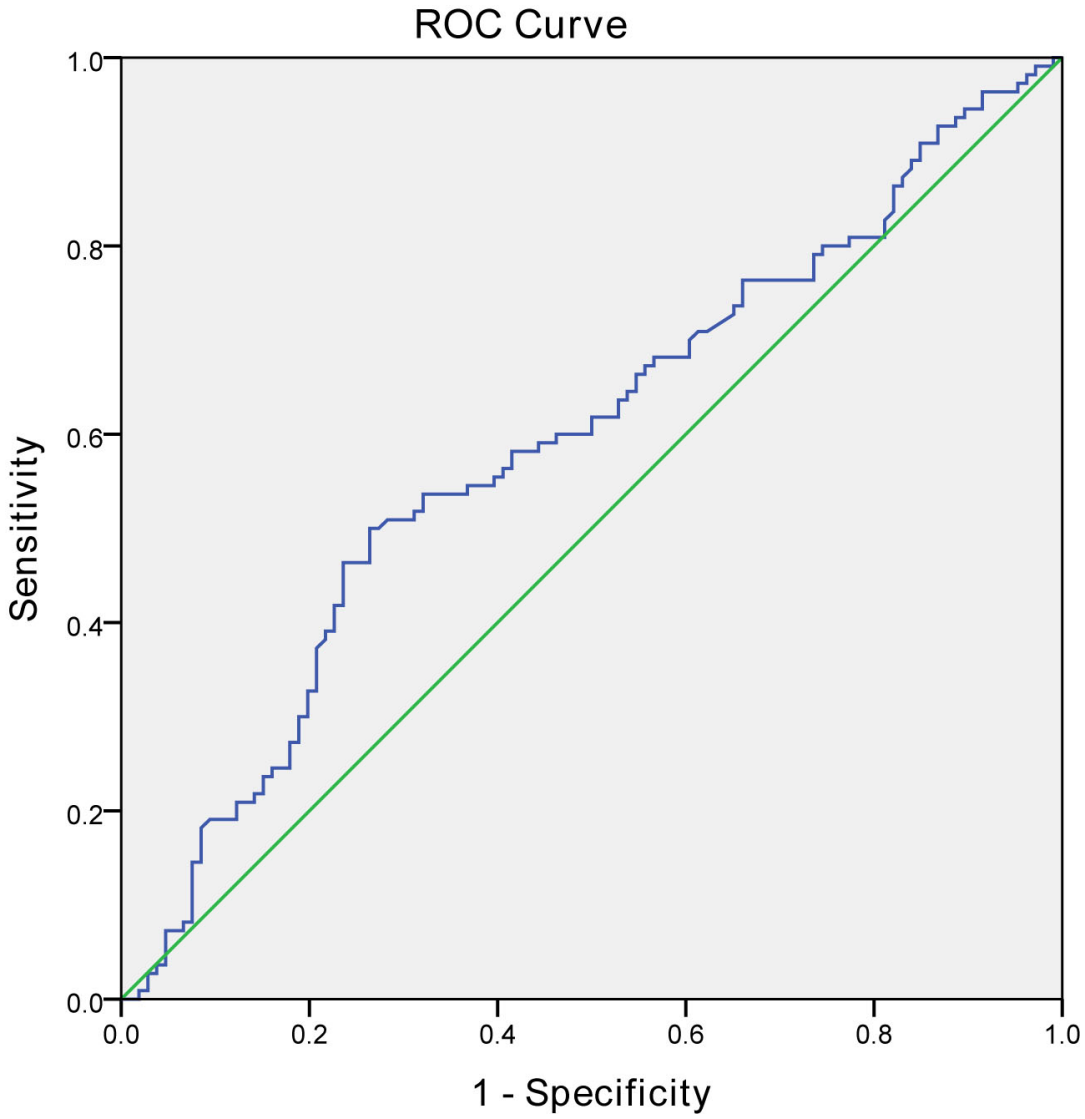
The ROC curve of MLR for the prediction of all-cause mortality in patients undergoing hemodialysis.

We also employed the ROC curve analysis to determine the diagnostic accuracy of MLR (shown in **Figure 2**). The results showed that MLR had predictive diagnostic value for all-cause mortality in patients undergoing hemodialysis. The diagnostic threshold was MLR >0.46 (AUC = 0.591, *P* = 0.039), with a sensitivity of 50.9% and a specificity of 73.6%.

### The correlation between MLR and clinical data

We analyzed the correlation between MLR and clinical data, shown in **Table 2**. Spearman’s analysis showed that MLR was positively correlated with BUN (*R* = 0.210, *P* = 0.002), WBC (*R* = 0.178, *P* = 0.009), and neutrophil (*R* = 0.237, *P* < 0.001).

**Table 2:** Correlation between MLR with the clinical data of the study population.

Variable	MLR
Age	R=0.090 P=0.188
Systolic blood pressure	R=-0.065 P=0.351
Diastolic blood pressure	R=-0.092 P=0.184
Diabetes	R=-0.013 P=0.845
SCr	R=0.026 P=0.703
BUN	R=0.210 P=0.002*
iPTH	R=-0.045 P=0.510
Serum iron	R=0.055 P=0.425
Cholesterol	R=0.132 P=0.080
Triglyceride	R=-0.129 P=0.089
WBC	R=0.178 P=0.009*
Platelets	R=0.070 P=0.303
Neutrophil	R=0.237 P<0.001*

SCr, serum creatinine; BUN, blood urea nitrogen; WBC, white blood cell; iPTH intact parathyroid hormone. *p<0.05. Values in bold represent statistically significant differences (p<0.05).

### MLR and cardiovascular and all-cause mortality

**Figure 3:**
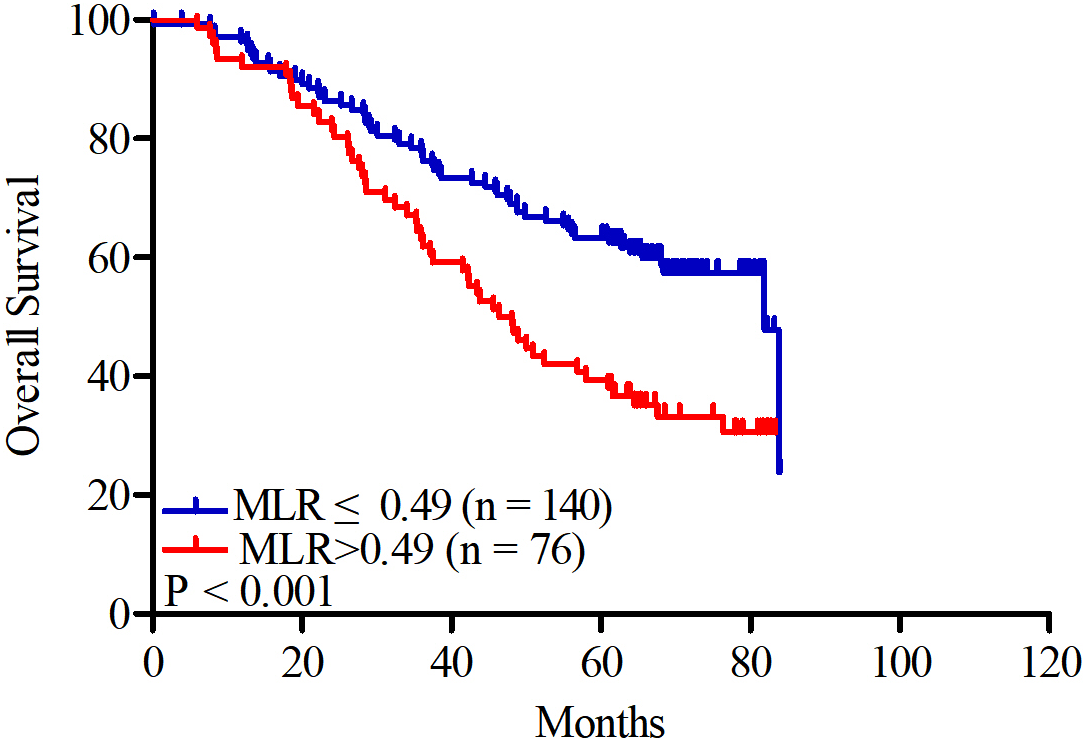
Kaplan–Meier curves for all-cause mortality in patients undergoing hemodialysis according to MLR.

A total of 110 patients (50.93%) died at the end of the study. In the low MLR group, the cumulative survival rates at 1, 3, and 5 years were 97.14%, 74.29%, and 63.57%, respectively; however, in the high MLR group, the rates were 92.11%, 55.26%, and 31.58%, respectively. Kaplan–Meier analysis showed that patients in the low MLR group present longer survival (64.08 ± 2.30 vs. 51.07 ± 3.12months, *P* < 0.001, shown in **Figure 3**).

The association between baseline characteristics and all-cause mortality is shown in **Table 3**. Univariate analysis showed that age (*P* < 0.001), diabetes (*P* < 0.001), albumin (*P* = 0.031), SCr (*P* < 0.001), cystatin (*P* = 0.015), MLR (*P* = 0.030), K (*P* = 0.035), P (*P* = 0.034), and iPTH (*P* = 0.023) were prognostic factors for overall survival in the total cohort. Multivariate Cox regression analysis showed that age (HR = 1.034, 95% CI: 1.017–1.051, *P* < 0.001), diabetes (HR = 2.126, 95% CI: 1.282–3.202, *P* = 0.002), and MLR (HR = 2.743, 95% CI: 1.137–6.615, *P* = 0.025) were factors significantly associated with a higher risk of all-cause mortality (**Table 3**).

**Table 3:** Univariate and multivariate analyses for all-cause mortality in the total cohort.

Variables	Univariate analysis HR (95% CI)	P value	Multivariate analysis HR (95% CI)	P value
Age (year)	1.047 (1.032-1.062)	<0.001*	1.034 (1.017-1.051)	<0.001*
Female sex	0.827 (0.554-1.234)	0.349		
Hypertension	1.378 (0.560-3.389)	0.464		
Diabetes	3.286 (2.196-4.917)	<0.001*	2.126 (1.282-3.202)	0.002*
Albumin (g/L)	0.955 (0.917-0.996)	0.031		
SCr (µmol/L)	0.999 (0.998-0.999)	<0.001*		
BUN (mmol/L)	0.996 (0.969-1.023)	0.757		
Uric acid (µmol/L)	0.998 (0.997-1.000)	0.055		
Cystatin (mg/L)	0.894 (0.811-0.986)	0.015*		
Triglyceride (mmol/L)	0.828 (0.641-1.069)	0.148		
Neutrophil (10^9^/L)	1.020 (0.925-1.126)	0.687		
Lymphocytes (10^9^/L)	0.856 (0.582-1.259)	0.430		
Monocytes (10^9^/L)	1.632 (0.787-3.384)	0.188		
Platelets (10^9^/L)	0.999 (0.997-1.002)	0.508		
Haemoglobin (g/L)	0.993 (0.983-1.004)	0.209		
NLR	1.032 (0.977-1.090)	0.263		
PAR	1.005 (0.925-1.092)	0.906		
PLR	1.000 (0.999-1.002)	0.814		
MLR	2.472 (1.094-5.587)	0.030*	2.743 (1.137-6.615)	0.025*
K (mmol/L)	0.767 (0.599-0.981)	0.035*		
Ca (mmol/L)	0.724 (0.380-1.380)	0.327		
P (mmol/L)	0.733 (0.550-0.976)	0.034*		
iPTH (pg/ml)	0.999 (0.998-1.000)	0.023*		
β2-MG	1.000 (0.975-1.026)	0.975		

SCr, serum creatinine; BUN, blood urea nitrogen; NLR, neutrophil-to-lymphocyte ratio; PLR, platelet-to-lymphocyte ratio; MLR, monocyte-to-lymphocyte ratio; iPTH, intact parathyroid hormone; β2-MG, β2-microglobulin. *p<0.05. Values in bold represent statistically significant differences (p<0.05).

The association between baseline characteristics and CVD mortality is shown in **Table 4**. Univariate analysis showed that age (*P* < 0.001), diabetes (*P* < 0.001), SCr (*P* = 0.026), and MLR (*P* = 0.033) were prognostic factors for overall survival in the total cohort. Multivariate Cox regression analysis showed that age (HR = 1.043, 95% CI: 1.016–1.071, *P* = 0.002), diabetes (HR = 2.490, 95% CI: 1.224–5.067, *P* = 0.012), and MLR (HR = 3.911, 95% CI: 1.135–13.476, *P* = 0.031) were factors significantly associated with a higher risk of CVD mortality (**Table 4**).

**Table 4:** Univariate and multivariate analyses for cardiovascular disease mortality in the total cohort.

Variables	Univariate analysis HR (95% CI)	P value	Multivariate analysis HR (95% CI)	P value
Age (year)	1.051 (1.028-1.075)	<0.001*	1.043 (1.016-1.071)	0.002*
Female sex	1.047 (0.571-1.920)	0.881		
Hypertension	1.331 (0.694-2.554)	0.390		
Diabetes	3.815 (2.028-7.177)	<0.001*	2.490 (1.224-5.067)	0.012*
Albumin (g/L)	0.949 (0.891-1.011)	0.107		
SCr (µmol/L)	0.999 (0.998-1.000)	0.026*		
BUN (mmol/L)	1.019 (0.980-1.060)	0.342		
Uric acid (µmol/L)	0.998 (0.996-1.001)	0.148		
Cystatin (mg/L)	0.974 (0.862-1.102)	0.678		
Triglyceride (mmol/L)	0.843 (0.575-1.238)	0.384		
Neutrophil (10^9^/L)	1.105 (0.961-1.271)	0.162		
Lymphocytes (10^9^/L)	0.855 (0.472-1.546)	0.603		
Monocytes (10^9^/L)	2.408 (0.934-6.211)	0.069		
Platelets (10^9^/L)	1.001 (0.998-1.005)	0.522		
Haemoglobin (g/L)	0.987 (0.972-1.003)	0.117		
NLR	1.045 (0.964-1.133)	0.289		
PAR	1.038 (0.925-1.165)	0.522		
PLR	1.001 (0.999-1.004)	0.218		
MLR	3.556 (1.110-11.390)	0.033*	3.911 (1.135-13.476)	0.031*
K (mmol/L)	0.810 (0.569-1.153)	0.242		
Ca (mmol/L)	0.885 (0.446-1.759)	0.728		
P (mmol/L)	0.782 (0.507-1.208)	0.268		
iPTH (pg/ml)	0.999 (0.998-1.000)	0.186		
β2-MG	1.002 (0.965-1.041)	0.900		

SCr, serum creatinine; BUN, blood urea nitrogen; NLR, neutrophil-to-lymphocyte ratio; PLR, platelet-to-lymphocyte ratio; MLR, monocyte-to-lymphocyte ratio; iPTH, intact parathyroid hormone; β2-MG, β2-microglobulin. *p<0.05. Values in bold represent statistically significant differences (p<0.05).

## Discussion

ESRD is strongly associated with elevated risks of morbidity, mortality, and healthcare costs, primarily driven by cardiovascular complications, infections, and multi-organ dysfunction due to progressive kidney failure (2). The most common form of kidney replacement therapy is dialysis, with hemodialysis comprising 89% of all dialysis procedures (9). The mortality rate of patients after initiation of dialysis is high, and the leading causes of mortality are CVD and infections. Microinflammation is a key factor for complications and mortality in hemodialysis (10). MLR can reflect systemic inflammation and serve as a reliable predictor of long-term survival in hemodialysis patients. Compared with traditional inflammatory indicators, MLR can be used as a cost-effective routine blood test parameter to identify high-risk hemodialysis patients (6).

In this study, we aimed to examine the association between MLR and the risk of CVD and all-cause mortality in patients initially receiving hemodialysis. We enrolled 216 patients initially receiving hemodialysis for at least 3 months, and the patients were divided into the low MLR group (<0.49) and the high MLR group (≥0.49). Our results showed that the levels of neutrophil and serum iron and the number of deaths were significantly higher in the high MLR group (*P* < 0.05). Spearman’s analysis showed that MLR was positively correlated with BUN (*P* = 0.002), WBC (*P* = 0.009), and neutrophil (*P* < 0.001). Kaplan–Meier analysis showed that patients in the low MLR group present longer survival. Multivariate Cox regression analysis showed that age, diabetes, and MLR (all *P* < 0.05) were factors significantly associated with a higher risk of CVD and all-cause mortality.

Inflammation, driven by multifactorial mechanisms, including uremic toxin accumulation, bioincompatible dialysis membranes, oxidative stress, metabolic dysregulation, and chronic infections, is very common in hemodialysis patients, resulting in increased morbidity and mortality (11). MLR, an easily obtainable inflammatory indicator, shows broad applications in oncology, infectious diseases, cardiovascular diseases, and nephrology. It demonstrates particular utility in prognostic stratification, therapeutic response prediction, and pathophysiological mechanism exploration (12–14). MLR can reflect systemic inflammation and chronic low-grade inflammation in hemodialysis patients, because elevated MLR indicates the activation of monocytes (pro-inflammatory cells) and the depletion of lymphocytes (anti-inflammatory cells) (15). Neutrophils and monocytes are important cells of the innate immune system. They are the most important cells responsible for increased immune response to uremic toxins and chronic contact with bio-incompatible membranes during hemodialysis treatment and can release cytokines and chemokines (10, 16). This can explain our results. In this study, we found that the level of neutrophil and the number of deaths were significantly higher in the high MLR group. Furthermore, Spearman’s analysis showed that MLR was positively correlated with BUN, WBC, and neutrophil. These data coincide with other data. Previous studies also found that neutrophil and MLR are biomarkers of inflammation in dialysis patients and were related to the prognosis of the patients (5, 15, 17, 18). Additionally, the high MLR group exhibited a pre-existing hyperinflammatory state prior to treatment, which may be associated with underlying conditions such as infections, chronic kidney disease, or metabolic disorders. Our study also found that the level of serum iron was significantly higher in the high MLR group. Previous studies found that serum iron was strongly associated with inflammation in hemodialysis patients, and iron overload could increase the risk of CVD in hemodialysis patients (19, 20). Therefore, neutrophil, serum iron, and MLR can indicate the inflammatory state in hemodialysis patients.

Our study found that MLR was an independent risk factor for cardiovascular and all-cause mortality in patients initially receiving hemodialysis. CVD is the leading cause of death in dialysis patients, especially in those who have just started hemodialysis treatment (10). Chronic low-grade inflammation plays a critical role in the pathogenesis of atherosclerosis, vascular calcification, and other etiologies of cardiovascular diseases (21). In dialysis patients, monocyte-derived inflammatory macrophages contribute to endothelial injury and atherosclerosis through adhesion to the vascular endothelium, release of pro-inflammatory mediators (e.g., IL-6, TNF-α), and induction of oxidative stress, thereby causing endothelial injury and thus being associated with atherosclerotic diseases and cardiovascular events (11). On the other hand, a low lymphocyte count (lymphopenia) is regarded as a risk factor for cardiovascular diseases and overall mortality (22, 23). This might explain why an increase in the MLR is associated with adverse clinical outcomes in hemodialysis patients. Previous studies also found that MLR can predict cardiovascular and all-cause mortality in dialysis patients (7). Wen et al. (18) found that the highest MLR tertile was significantly associated with an increase in CVD and all-cause mortality in peritoneal dialysis patients. Han et al. (17) found that the combination of MLR and other inflammatory markers can predict the CVD prognosis in maintenance hemodialysis patients. Therefore, MLR can be used as a tool for clinicians to evaluate the prognosis of hemodialysis patients.

Our study also found that age and diabetes were factors significantly associated with a higher risk of cardiovascular and all-cause mortality in patients initially receiving hemodialysis. In elderly patients, the function of T cells declines and the immune response weakens (24). When combined with diabetes or hemodialysis, the inflammatory response increases, and the risk of infection significantly increases (25). In patients with diabetes, hyperglycemia can further lead to the accumulation of pro-inflammatory substances, the activation of monocytes, and the release of inflammatory mediators, thereby causing infections and a decline in immune function (26). Qiu et al. (27) found that in diabetes patients with CKD in the intensive care unit, high MLR was significantly associated with increased risk of 90-day all-cause mortality. Therefore, age and diabetes also have an impact on the poor prognosis of hemodialysis patients.

There are still several limitations in our study. First, this is a single-center retrospective study; the number of patients and events is limited, and there may be inherent biases. Second, other traditional inflammatory markers, such as CRP, interleukin, and TNF, were not included in our study because they are expensive and not routinely measured in hemodialysis patients. Third, we did not explore the mechanisms and the inflammatory pathway in hemodialysis patients. Further studies should be carried out to explore these results.

## Conclusions

In conclusion, our results revealed that high MLR values are an independent risk factor for cardiovascular and all-cause mortality in hemodialysis patients, especially in the elderly and those with a history of diabetes. MLR is a straightforward and inexpensive indicator to reflect systemic inflammation status. Our findings suggested that MLR can be used as a tool for clinicians to evaluate the prognosis of hemodialysis patients. Further research should focus on the inflammatory pathway to reduce inflammation and improve the prognosis of hemodialysis patients.

## Data Availability

Publicly available datasets were analyzed in this study. This data can be found here: The datasets are available from the corresponding author on reasonable request.
